# Hospital Workload for Weapon-Wounded Females Treated by the International Committee of the Red Cross: More Work Needed than for Males

**DOI:** 10.1007/s00268-017-4160-y

**Published:** 2017-08-09

**Authors:** Peter Andersson, Måns Muhrbeck, Harald Veen, Zaher Osman, Johan von Schreeb

**Affiliations:** 1 Center for Teaching and Research in Disaster Medicine and Traumatology, University Hospital, 581 85 Linköping, Sweden; 2 Department of Surgery, University Hospital, 581 85 Linköping, Sweden; 3 Department of Clinical and Experimental Medicine, Linköping University, 581 83 Linköping, Sweden; 4 Department of Surgery, Vrinnevi Hospital, 603 79 Norrköping, Sweden; 5 International Committee of the Red Cross, 19 Avenue de la Paix, 1202 Geneva, Switzerland; 6 Department of Public Health Sciences, Karolinska Institutet, 171 65 Solna, Sweden

## Abstract

**Background:**

Civilians constitute 33–51% of victims in armed conflicts. Several reports on civilian injuries exist, but few have focused on injuries afflicting females. We analyzed routinely collected data on weapon-related injuries from the International Committee of the Red Cross (ICRC) hospital in northwestern Pakistan in order to define injury patterns and types of surgical treatment for females.

**Methods:**

A total of 3028 patient files (376 females) from consecutively admitted patients to the ICRC-hospital in Peshawar from February 2009 to May 2012 were included. Information regarding injury-mechanism, time since injury, vital parameters at admission, type of injury, treatment and basic outcome was extracted from the files and analyzed. Comparisons between gender and age-groups were done by cross-table analyses or nonparametric tests.

**Results:**

Females were younger than males (20 vs. 25 years), arrived sooner after injury (24 vs. 48 h) (*p* < 0.001 for both) and were victims of bombs and missiles more frequently (64.4 vs. 54.6%) (*p* < 0.001). Vital parameters such as systolic blood pressure (110 vs. 113 mmHg) and pulse rate (100 vs. 86) were more affected at admission (*p* < 0.001 for both). Females were subjected to surgery (83.0 vs. 77.4%) (*p* < 0.05) and were given blood transfusions more often (18.8 vs. 13.6%) (*p* < 0.01). No differences in amputations or in-hospital mortality were found.

**Conclusions:**

Females treated at the ICRC-hospital in northwestern Pakistan are markedly affected by indiscriminate weapons such as bombs and missiles. Their average consumption of surgery is greater than for males, and this might be relevant in planning for staffing and facility needs in similar contexts.

## Introduction

Civilians constitute 33–51% of victims in regions of armed conflict [1, 2]. Several reports on civilian injuries from such regions indicate that the highest proportions of injuries of women and children are caused by indiscriminate weapons such as mortars and missiles fired from a distance [3, 4]. There are, however, few reports focusing on injuries afflicting females in regions of armed conflict. We have not identified any report that specifies the types of injuries or the workload that these injuries impose on needs at health care facilities. To fill this research gap we decided to analyze routinely collected surgical hospital data on weapon-related injuries from the International Committee of the Red Cross (ICRC). The ICRC is an organization working in conflict-areas independently running or otherwise supporting hospitals providing surgery with limited resources for free for the weapon-wounded. Since 1991 the ICRC administers a database where all weapon-wounded patients admitted to hospitals operated or supported by the organization are registered. At present there are more than 56,000 patients in the database for whom epidemiological and surgical treatment information on weapon-injuries inflicted in various regions of armed conflict is available.

## Aim of the study

To define patterns of injury and type of surgical treatment for injured females admitted to the ICRC-hospital in northwestern Pakistan.

### Patients and methods

A total of 3028 patient files (376 females) from patients consecutively admitted to the ICRC-hospital in Peshawar from February 2009 to May 2012 were included. The dates were the starting and ending dates for the ICRC-operated hospital which was located within the city. Only weapon-related injuries were treated in the hospital during the study-period. Patients’ injuries were mainly generated from an ongoing asymmetric armed conflict characterized by the use of explosives, grenades, attacks by gunmen and remote-controlled aerial bombings on both sides of the Pakistani–Afghanistan border [5]. The distance between Peshawar and the Afghanistan border is 60 km. Injuries were also generated from attacks such as suicide bombings taking place within the city of Peshawar or in the surrounding area. Patients were not asked at admission whether they were civilians or combatants. The ICRC-hospital was, together with some small facilities run by non-governmental organizations, one of few hospitals in the area providing surgery for weapon-wounded also of afghan origin completely free of charge. Several public hospitals provided initial surgical care for free in the area, but these were mainly strictly located to Peshawar and were difficult to access for others than Pakistanis.

Information regarding sex, age, time since injury, vital parameters at admission including level of consciousness, mechanism of injury, type of treatment, body-region affected and outcome parameters such as discharge with or without follow-up as well as in-hospital mortality was prospectively entered into an Excel-database mainly by one of the authors (ZO).

We identified injury patterns from injury-mechanism and body-region affected as well as treatment practices for females in comparison to males treated at the hospital. In keeping with previous reports from ICRC patient-cohorts, patients younger than 16 years of age were classified as boys or girls and patients 16 years or older as men or women. Accordingly, all females together with all males aged less than 16 or more than 49 were classified as civilians [2, 6, 7]. During the study-period, 376 females (median age 20, range 0.3–84 years) were treated along with 2633 males (24, 1–80). Data regarding sex were missing for 19 patients.

### Statistical methods

Statistical analysis was done using IBM SPSS Statistics software version 23 (IBM Corporation, Armonk, NY, USA). Types of surgical procedures and blood transfusions are given as percentages of patient numbers, i.e., if a patient had serial laparotomies or multiple transfusions only one such was counted. Consequently, repeat amputations on the same limb were counted as one and identified by the last level of amputation. Values are given as mean (SD) or median (IQR) when applicable. Comparisons between groups were done by cross-table analyses (*χ* ^2^-test) or nonparametric tests (Mann–Whitney U) when appropriate. Significance level was set to *p* < 0.05 two-tailed test.

## Results

**Fig. 1:**
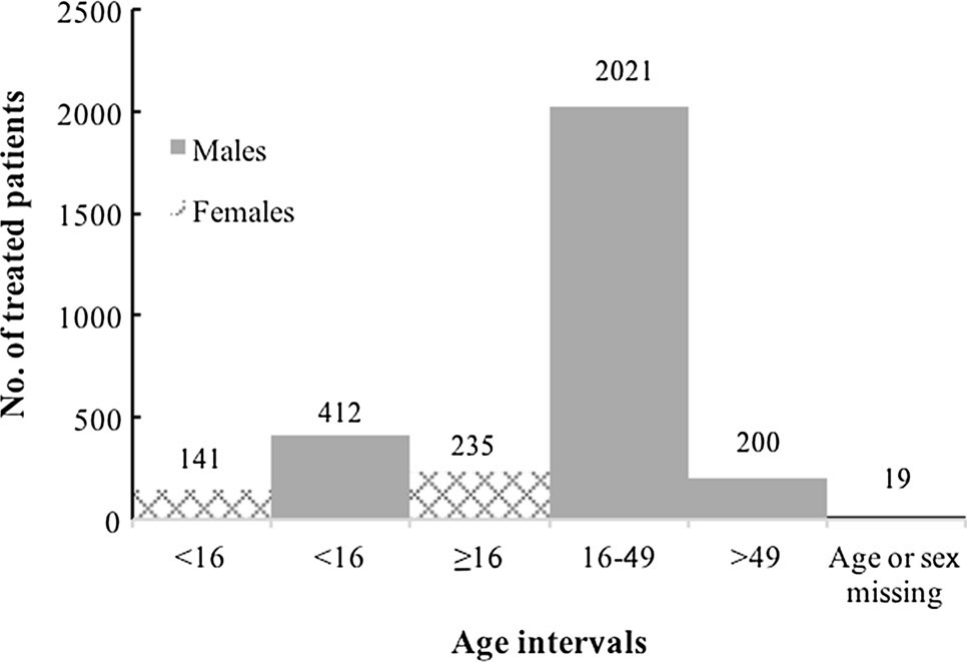
Age distribution of patients treated at ICRC’s hospital for weapon-wounded in Peshawar, Pakistan, 2009–2012

Data from totally 3028 patient files were analyzed. The distribution of patients according to sex- and age-groups is shown in Fig. 1. Females constituted 12.4% of all patients and civilians 32.8%. Girls (*n* = 141) made up slightly more than one-third of all females, whereas boys (*n* = 412) constituted less than one-fifth of all males. Females were younger than males; median age 20 for females, 25 for males, interquartile ranges (IQR) 23 females, 14 males (*p* < 0.001) and arrived sooner after injury than males; median-time since injury 24 h for females and 48 h for males (IQR 88; 177) (*p* < 0,001). A total of 206 (54.8%) female patients reached hospital within 24 h compared to 1055 (40.1%) male patients. Vital parameters at admission such as systolic blood pressure, pulse rate and respiratory rate were more severely affected in females than in males (*p* < 0.001 for all). Level of consciousness was equally affected in females and males (Table 1). Females were victims of bombs and missiles more frequently than males and more often sustained concomitant burns from subsequent fire, whereas males suffered from bullet-injuries to a greater extent (Table 2). Both sexes were equally affected by antipersonnel mines (APM). Table 3 shows the types of surgery needed for females and males, respectively, and also for age subgroups. The female group was subjected to any type of surgery of the skull, chest and abdomen more often than the male group (*p* < 0.05) as it was for any kind of surgery (*p* < 0.05) as well as for blood transfusions (*p* < 0.01). Girls, as a subgroup, were subjected to surgery of the skull, chest and abdomen to a greater extent than were boys (*p* < 0.05). However, the need for any type of surgery or blood transfusions did not differ between these two subgroups. No difference between sexes was found regarding the need for amputation or for in-hospital mortality.

**Table 1:** Vital parameters at hospital admission in females and males treated at ICRC’s hospital for weapon-wounded in Peshawar, Pakistan, 2009–2012

Vital parameters	All females		Missing	All males		Missing	*p* Value
	*n* = 376			*n* = 2633			
Systolic blood pressure	110	(18.1)	10	113	(16.8)	60	*p* < 0.001
Pulse rate	100	(20.3)	182	86	(16.6)	1123	*p* < 0.001
Respiratory rate	22	(4.4)	182	21	(3.8)	1139	*p* < 0.001
Glasgow coma scale	14.6	(1.4)	177	14.7	(1.5)	1118	*p* = 0.14

Figures are mean (SD)

**Table 2:** Comparison of mechanism of injury for all females and males treated at the ICRC’s hospital for weapon-wounded in Peshawar, Pakistan, 2009–2012

Mechanism of injury	All females		All males		*p* Value
	*n* = 376		*n* = 2633		
Bomb or missile	242	(64.4)	1437	(54.6)	*p* < 0.001
Fire^a^	19	(5.0)	36	(1.4)	*p* < 0.001
Bullet	107	(28.5)	1012	(38.4)	*p* < 0.001
Antipersonnel mine	16	(4.3)	150	(5.7)	*p* = 0.25
Other	7	(1,9)	25	(0,9)	*p* < 0.01
Missing	4	(1.1)	9	(0.3)	–

Figures are numbers, percentages are given within brackets

^a^ Fire constitutes a subgroup within the group bomb or missile

**Table 3:** Distribution of surgical procedures at the ICRC’s hospital for weapon-wounded in Peshawar, Pakistan, 2009–2012

Type of procedure	Girls < 16 years	Women ≥ 16 years	All females	Boys < 16 years	Men 16–49 years	Men > 49 years	All males	*p* Value all females versus all males	*p* Value all girls versus all boys
	*n* = 141	*n* = 235	*n* = 376	*n* = 412	*n* = 2021	*n* = 200	*n* = 2633		
Craniotomy	7 (5.0)	5 (2.1)	12 (3.2)	12 (2.9)	36 (1.8)	1 (0.5)	49 (1.9)	0.09	0.38
Thoracotomy	2 (1.4)	2 (0.9)	4 (1.1)	5 (1.2)	14 (0.7)	1 (0.5)	20 (0.8)	0.54	0.80
Chest-tube insertion	13 (9.2)	15 (6.4)	28 (7.4)	23 (5.6)	127 (6.3)	16 (8.0)	166 (6.3)	0.38	0.19
Laparotomy	14 (9.9)	26 (11.1)	40 (10.6)	26 (6.3)	196 (9.7)	8 (4.0)	230 (8.7)	0.23	0.21
Any type of surgery head/chest/abdomen	30 (21.3)	42 (17.9)	72 (19.1)	55 (13.3)	318 (15.7)	22 (11.0)	395 (15,0)	<0.05	<0.05
Arm-amputation	1 (0.7)	2 (0.9)	3 (0.8)	10 (2.4)	33 (1.6)	0 (0)	43 (1.6)	0.22	0.36
Above-knee amputation	5 (3.5)	9 (3.8)	14 (3.7)	12 (2.9)	52 (2.6)	11 (5.5)	75 (2.8)	0.35	0.93
Below-knee amputation	5 (3.5)	15 (6.4)	20 (5.3)	22 (5.3)	68 (3.4)	14 (7.0)	104 (3.9)	0.21	0.53
Any type of amputation	9 (6.4)	24 (10.2)	33 (8.8)	42 (10.2)	139 (6.9)	25 (12.5)	206 (7.8)	0.52	0.24
Any type of surgery soft-tissues^a^	93 (66.9)	174 (74.0)	267 (71.0)	276 (67.0)	1371 (67.8)	151 (75.5)	1798 (68.3)	0.29	0.90
Any type of surgery all kinds	111 (78.7)	201 (85.5)	312 (83.0)	315 (76.5)	1555 (76.9)	167 (83.5)	2037 (77.4)	<0.05	0.66
Any blood transfusion	26 (18.4)	44 (18.7)	70 (18.8)	73 (17.7)	253 (12.5)	28 (14.0)	354 (13.6)	<0.01	0.95
Mortality	10 (7.1)	14 (6.0)	24 (6.4)	20 (4.9)	89 (4.4)	12 (6.0)	121 (4.6)	0.13	0.43

Figures are numbers, percentages are given within brackets. Some patients have been subjected to several procedures but at different sites which results in more procedures than patients and a sum of percentages more than 100

^a^ Debridement, delayed primary closure, split skin graft or burn care

## Discussion

To our knowledge this is the first report focusing on injuries and surgery in females in the context of an asymmetric armed conflict such as the ongoing conflict in northwestern Pakistan and bordering Afghanistan where a substantial inequality in military power exists between the warring parties [5]. In contrast with recent reports from other areas torn by armed conflict where females constitute up to 43% of the patients treated the percentage of females in this study was much smaller, 12.4% [8]. This difference may be explained by the fact that treatment at ICRC-facilities such as the hospital in Peshawar is strictly limited to weapon-related injuries. The low percentage of females treated corresponds quite well, however, with percentages reported from an Afghanistan trauma-center operating in a similar setting where 15% of patients treated were females [9]. Females were injured by fragments from mortars and bombs significantly more often than males. Most likely these injuries were sustained in public places or in domestic settings. Recent reports from Syria reveal a similar pattern of injuries among women and children with the highest death tolls caused by fragments emanating from air bombardments and ground explosives [1]. These weapons are indiscriminate by nature affecting civilians to a proportionally greater extent than combatants as shown by data from Kabul as far back in time as 25 years and also from Iraq between 2003 and 2008 [3, 10]. In the present study the data for females as representatives of the civilian group confirm these earlier findings. It might be claimed, however, that some of the difference between females and males might be due to a selection of less seriously injured males reaching hospital as indicated by their significantly later arrival after injury. Longer transportation distances might result in a greater proportion of pre-hospital deaths from fragmented munitions among males. Considering also the greater affection of vital parameters in females it seems most likely that females represent a different cohort of weapon-wounded treated in this setting than the cohort of males. Apparently the female cohort comes from areas closer to the health facility than does the male cohort, and also, consists of a larger proportion of children and of victims in worse condition upon arrival than the male cohort. The more affected vital parameters can to some part be explained by the larger proportion of children below 16 years of age among females but not completely since these differences, except for systolic blood pressure, persist also even after removal from the analysis of all data for children younger than 16 years of age (not shown). The differences in vital parameters, particularly blood pressure and respiratory rate, might seem to be negligible as seen from an individual clinical perspective, but on a group level their collective values signal a true poorer physical condition in females.

Injuries caused by APM were relatively uncommon among both females and males. The figure of 5.4% afflicted in the entire study-population contrasts sharply with figures reported from the beginning of the 1990s when 48% of all weapon-wounded from the same area in northwestern Pakistan and neighboring Afghanistan were injured as a result of mines [11]. This is likely a result of the global work against landmines by both governmental and non-governmental bodies that resulted in the Ottawa treaty endorsed by a majority of states in 1997 prohibiting their use [12]. In-hospital mortality, although slightly higher among females, did not differ significantly between sexes, and the total figure of 4.8% is in line with other studies from the same geographical area reporting rates of 2.5–7.0% [9, 13, 14]. Females consumed more hospital resources per person than did males since they were subjected to more surgical procedures involving the skull, thorax and abdomen, had more procedures as a whole, and given more blood transfusions. The explanation is probably that, as already mentioned, females represent a group of weapon-wounded coming from areas closer to the health facility and thereby are likely to survive more serious injuries than males during the time between being wounded and the time of arrival. Another explanation might be that females injured at a greater distance from the ICRC-hospital even with less severe injuries are never transferred to the hospital as already suggested by Coupland and Korver [6] in a report from the same area 1991. The reason for this might be that were there fewer transporters than needed and that males were given priority.

This study has limitations. It only covers the weapon-wounded females treated at one facility. Many weapon-wounded patients and patients affected by terror-attacks are taken care of by public hospitals or other agencies operating in the area. Unfortunately the proportion of patients treated at the ICRC-hospital could not be determined since coordinated data on the total numbers of weapon-wounded in the area are lacking. Moreover, although data are prospectively collected, no particular study objective was identified prior to collection which might have affected quality of data. As the objective of this study was to describe the ICRC’s experience of surgical treatment of the war wounded in northwestern Pakistan with focus on females, no patients were excluded. Therefore, a limited number of patients were included who had injuries older than three months. This most likely falsely affected mortality in a positive manner compared to other studies.

## Conclusions

At the ICRC-hospital in Peshawar, Pakistan, where exclusively war-wounded patients were treated, females were in a minority. They were more likely than males to be injured by indiscriminate weapons such as bombs and missiles. Female patients arrived sooner after injury, in a worse clinical condition than males and, on average, had a higher requirement for blood and surgical services than males. This knowledge might be relevant in planning for staffing and facility needs in similar contexts.
